# Utilization of breast MRI and breast MRI-guided biopsy in clinical practice: results of a survey in Québec and France

**DOI:** 10.1186/s13244-020-00886-3

**Published:** 2020-07-01

**Authors:** Benoît Mesurolle, Simon Sun, Michelle Zhang

**Affiliations:** 1 Centre République, ELSAN, 99 avenue de la République, BP 304, 63023 Clermont-Ferrand Cedex 2, France; 2 Breast Clinic, McGill University Health Center, Royal Victoria Hospital, 1001 Decarie Boulevard, Montreal, QC H4A 3 J1 Canada

**Keywords:** Breast MRI, Breast MRI-guided biopsy, Survey, Healthcare system, France, Québec

## Abstract

**Background:**

To investigate the practice regarding breast MRI exams and breast MRI-guided biopsies in two countries with different health care systems, France and Québec. A 12-item questionnaire was distributed online among radiologists from France and Québec, attempting to determine: demographic characteristics and breast MRI diagnostic and MRI-guided practices (indications, workload, availability, and waiting time assessment).

**Results:**

One hundred and seventy radiologists (France, 132 respondents (28.5%); Quebec, 38 respondents (35.2%)) participated in the survey, most of them based in non-academic centers. Thirty-eight percent of Quebec and 2.3% of French radiologists did not perform breast MRI in their daily practice. Nearly 50% of French and Quebec respondents interpreted 1–10 breast MRI exams per week. Decision-making factors of preoperative MRI were similar in both countries (pathology, age, and breast density), with a heavier emphasis placed on the surgeon’s opinion in Quebec (47.8% versus 21.8% (*p* = 0.009)). Quebec demonstrated a higher waiting time than France (1–2 weeks in 40% versus less than 1 week in 40%). MRI-guided breast biopsies (less than 5 MRI-guided biopsies per week) were being performed by a minority of the respondents (36% in France and 43% in Québec).

**Conclusion:**

Most of radiologists performing breast MRIs work in non-academic institutions in both countries. Waiting time is higher in Quebec, but most of preoperative breast MRIs are performed within 3 weeks in both countries. The surgeon plays an important role in recommending preoperative MRI in Quebec. MRI-guided breast biopsies are not widely available in both countries.

## Key points

- Half of French and Quebec radiologists interpret 1–10 breast MRI exams per week
- MRI-guided breast biopsies are not widely available in both countries
- Quebec demonstrates a higher waiting time than France for breast MRI exams
- Most of preoperative breast MRIs are performed within 3 weeks in both countries
- Surgeon’s opinion influences recommendation of preoperative MRI in Quebec

## Background

While mammography and breast ultrasound remain the standard breast imaging modalities, breast magnetic resonance imaging (MRI) has also become important for the detection of breast carcinoma [1, 2]. Recently, its indications have dramatically increased, including screening, diagnosis, and staging [2]. However, breast MRI indications vary among radiologists and among hospitals, given the general lack of consensus [3], and its use as a preoperative staging tool remains a controversial topic [4].

In particular, breast MRI carries the risk of potentially lengthening surgical waiting times due to its propensity to create the need for second-look ultrasounds, with or without associated biopsy, along with increasing mastectomy rates [5–7].

Therefore, the purpose of the present study was to identify practice trends and opinions concerning breast MRI and MRI-guided biopsies in two different health care systems, one in a European (France) and the other in a North American country (Province of Quebec/Canada).

## Methods

From September 2015 to September 2016, a web-based survey was conducted with members of the SCFR (“Société Canadienne Française de Radiologie”, Québec, Canada) and SIFEM (“Société d’Imagerie de la Femme”, France) targeting radiologists involved in breast imaging. Names and e-mail addresses were obtained from the publicly available membership lists of these two radiological professional organizations. The web-based survey was administered via SurveyMonkey. Radiologists were contacted and asked to voluntarily complete an anonymous survey within 4 weeks; a single follow-up e-mail was sent a few weeks later to remind radiologists of the ongoing survey. A 12-item questionnaire was designed in which respondents were asked to choose a single best response for each question, except for one instance where multiple choices could be made (Table 1).

**Table 1:**
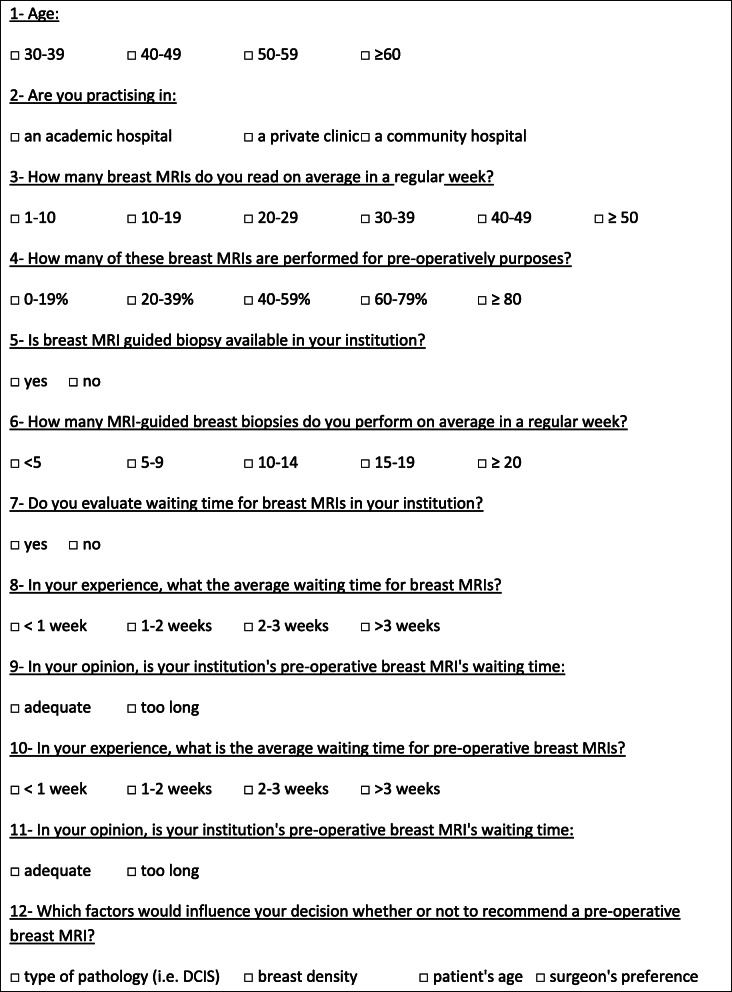
breast MRI survey

The survey was comprised of four sections (Table 1): (1) personal practice characteristics (e.g., physician age and type of practice), (2) characteristics of the breast MRI exams (e.g., preoperative MRI indication, factors influencing their recommendation), (3) availability of MRI-guided breast biopsies and their frequency, and (4) waiting time of breast MRI in their respective institutions. All questions and answers are detailed in Tables 2, 3, 4, 5, and 6.

**Table 2:** General information

	Québec		France		*p* value *χ*^**2**^ test
	Proportions	95% CI	Proportions	95% CI	
**Age (years)**					
**< 30**	0% (0/38)	[0–0]	0.8% (1/132)	[0–2.3]	0.590
**30–39**	18.4% (7/38)	[5.5–31.3]	18.9% (25/132)	[12.2–25.7]	0.943
**40–49**	47.4% (18/38)	[30.7–64]	28.8% (38/132)	[22–36.6]	0.032
**50–59**	26.3% (10/38)	[11.6–41]	37.1% (49/132)	[28.8–45.5]	0.218
**≥ 60**	7.9% (3/38)	[1.1–16.88]	14.4% (19/132)	[8.3–21.5]	0.293
**Academic hospital**	26.3% (10/38)	[11.6–41]	16.7% (22/132)	[10.2–23.1]	0.180
**Private clinic**	63.2% (24/38)	[47.1–79.2]	22.2% (28/132)	[14.1–28.3]	< 0.0001
**Community hospital**	10.5% (4/38)	[0.3–21.7]	62.1% (82/132)	[53.7–70.5]	< 0.0001

Note—Data in parentheses are ratio

**Table 3:** Breast MRI examinations

	Québec		France		*p* value *χ*^**2**^ test
	Proportions	95% CI	Proportions	95% CI	
**Number of examinations per week**					
**None**	37.8% (14/37)	[22.4–54.2]	2.3% (3/128)	[0.3–5]	< 0.0001
**1–10**	46% (17/37)	[29.1–62.8]	47.7% (61/128)	[38.9–56.4]	0.854
**10–19**	2.7% (1/37)	[2.8–8.2]	27.3% (35/128)	[19.5–35.2]	0.001
**21–29**	5.4% (2/37)	[2.2–13.1]	10.2% (13/128)	[4.8–15.5]	0.376
**≥ 30**	8.1% (3/37)	[1.1–17.3]	12.5% (16/128)	[6.7–18.3]	0.461
**Number of preoperative examinations**					
**0–24%**	61.9% (13/22)	[39.2–84.6]	41% (50/122)	[32.1–49.8]	0.096
**25–49%**	19% (4/22)	[0.7–37.4]	36.9% (45/122)	[28.2–45.6]	0.139
**50–74%**	4.8% (1/22)	[5.2–14.7]	13.1% (16/122)	[7.0–19.2]	0.468
**≥ 75%**	4.8% (1/22)	[5.2–14.7]	4.1% (5/122)	[7.0–19.2]	1.000
**Unsure**	9.5% (2/22)	[4.2–23.2]	4.9% (6/122)	[1.0–8.8]	0.396

Note—Data in parentheses are ratio

**Table 4:** Breast MRI-guided biopsies

	Quebec		France		*p* value *χ*^**2**^ test
	Proportions	95% CI	Proportions	95% CI	
**Breast MRI-guided biopsy availability**					
**Yes**	42.9% (9/22)	[19.8–65.9]	36.1% (44/122)	[27.4–44.7]	0.552
**Number of breast MRI-guided biopsies per week**					
**< 5**	100% (9/9)	[100–100]	97.7% (43/44)	[93.1–100]	0.648

Note—Data in parentheses are ratio

**Table 5:** Waiting time audit and estimated waiting time

	Québec		France		*p* value *χ*^**2**^ test
	Proportions	95% CI	Proportions	95% CI	
**Auditing waiting time**					
**Yes**	80% (16/21)	[60.8–99.2]	68.1% (81/119)	[59.6–76.6]	0.282
**Average waiting time breast MRI**					
**< 1 week**	0% (0/21)	[0–0]	16.8% (21/119)	[10–23.6]	0.048
**1–2 weeks**	5% (1/21)	[5.5–15.5]	44.6% (53/119)	[35.5–53.6]	0.001
**2–3 weeks**	25% (5/21)	[4.2–75.8]	25.2% (30/119)	[17.3–33.1]	0.984
**> 3 weeks**	55% (11/21)	[31.1–78.9]	12.6% (15/119)	[6.5–18.7]	< 0.0001
**Unsure**	15% (3/21)	[2.1–32.1]	0.8% (1/119)	[0.8–2.5]	< 0.0001
**Waiting time breast MRI opinion**					
**Adequate**	68.4% (13/19)	[45.4–91.4]	72 % (85/118)	[63.8–80.2]	0.746
**Too long**	31.6% (6/19)	[8.6–54.6]	28 % (33/118)	[19.7–36.2]	0.746
**Average waiting time preoperative breast MRI**					
**< 1 week**	5% (1/21)	[5.5–15.5]	40.3% (48/119)	[31.4–49.3]	0.002
**1–2 weeks**	40% (8/21)	[16.5–63.5]	52.1% (62/119)	[43–61.2]	0.317
**2–3 weeks**	30% (6/21)	[8–52]	6.7% (8/119)	[2.2–11.3]	0.001
**> 3 weeks**	10% (2/21)	[4.4–24.4]	0% (0/119)	[0–0]	0.001
**Unsure**	15% (3/21)	[2.1–32.1]	0.9% (1/119)	[0.8–2.5]	< 0.0001
**Waiting time preoperative breast MRI opinion**					
**Adequate**	85% (17/21)	[67.8–102.1]	81.5% (97/119)	[74.4–88.6]	1.000
**Too long**	15% (3/21)	[2.1–32.1]	18.5% (22/119)	[11.4–25.6]	1.000

Note—Data in parentheses are ratio

**Table 6:** Most important factors influencing your decision as to whether or not to recommend a preoperative breast MRI? (select all applicable choices)

	Québec		France		*p* value *χ*^**2**^ test
	Proportions	95% CI	Proportions	95% CI	
**Pathology (i.e., invasive vs. DCIS)**	78.3% (18/23)	[60–96.5]	87.2% (109/125)	[81.3–93.1]	0.259
**Patient age**	52.2% (12/23)	[30.1–74.3]	62.4% (78/125)	[53.8–71.0]	0.356
**Breast density**	47.8% (11/23)	[25.7–69.9]	46.4% (58/125)	[37.5–55.3]	0.900
**Surgeon’s preference**	47.8% (11/23)	[25.7–69.9]	21.8% (26/125)	[13.6–28.0]	0.006
**MRI availability**	13 % (3/23)	[1.85–27.9]	4.8% (6/125)	[1–8.6]	0.128
**Other**	13 % (3/23)	[1.85–28]	22.6% (27/125)	[14.3–28.9]	0.348

Note—Data in parentheses are ratio

### Breast MRI indications and numbers (questions 3, 4, and 12)

The purpose of these questions was to determine the number of breast MRI exams performed per week and the proportion of preoperative breast MRI in their practice. The reasons justifying their recommendations of breast MRI were also investigated.

### MRI-guided biopsies (questions 5 and 6)

The purpose of these questions was to determine if radiologists had the possibility of performing MRI-guided biopsies in their institution, and if so, to evaluate the number of biopsies performed per week.

### Waiting time breast MRI (questions 7, 8, 9, 10, and 11)

The purpose of these questions was to assess the waiting time for breast MRI in the institution of the responding radiologists. Breast MRI waiting time was defined as time from initiation of the request for breast MRI to the date of the actual breast MRI.

### Statistical analysis

Descriptive results were summarized in tables and presented as counts and percentages with 95% confidence interval. *χ*^2^ tests were used for categorical variables. All tests were two-sided, with the alpha significance level set at less than 0.05. Data was analyzed using SPSS (version 21.0, released 2011, Armonk, NY: IBM Corp).

## Results

### Response rate

In France, 132 of 463 radiologists (28.5%) responded, whereas in Quebec, 38 out of 108 radiologists responded (35.2%).

### Demographics (questions 1 and 2)

The characteristics of the study population are shown in Table 2. Most of the responding radiologists were “senior” radiologists, 74% aged between 40 and 59 in Québec and 66% aged between 40 and 59 in France. The three types of surveyed hospitals included academic medical centers, private clinics, and local community hospitals. Most radiologists were employed in non-academic institutions (i.e., general hospitals in Quebec, and private institutions in France).

### Breast MRI number, indications, and factors influencing recommendation of preoperative breast MRI (questions 3, 4, and 12) (Tables 3 and 6)

In Québec, 38% of respondents were not performing any breast MRI, whereas 49% performed between 1 and 21 breast MRI exams per week (mostly 1–10). In France, 75% of respondents performed between 1 and 19 MRI exams per week. There were significantly more radiologists in France performing between 10 and 19 breast MRI examinations per week than in Québec (27.3% versus 2.7%, *p* = 0.001). For the majority of radiologists in both countries, preoperative breast MRI exams were not the main clinical indication (representing less than 25% of breast MRI exam indications for 41% of respondents in France and 61.9% in Québec).

In both countries, the 3 most important decision-making factors for performing a preoperative breast MRI were found to be the pathologic features of breast carcinoma, followed by age and breast density. In Québec, the surgeon’s preference was a vital factor for 47.8% of the performed breast MRIs, as opposed to 21.8% in France (*p* = 0.006) (Table 6).

### MRI-guided biopsies (questions 5 and 6) (Table 4)

MRI-guided breast biopsy was not being performed at most of the surveyed radiological practices (64% in France and 57% in Québec). And when MRI-guided biopsy was an available procedural option, they were being performed less than 5 per week.

### Waiting time breast MRI (questions 7, 8, 9, 10 and 11) (Table 5)

In both countries, most facilities kept track of their MRI waiting time, with up to 68% of institutions in France and 80% in Québec performing audits. In Québec, waiting time was greater than 3 weeks in 55% of respondents (*p* < 0.001) whereas it was never reported as less than 1 week. In France, waiting time was between 1 and 2 weeks for 44.5% of respondents (*p* = 0.001) and was less than 1 week for 16.8% of respondents. In both countries, this waiting time was considered appropriate for most respondents.

With respect to preoperative breast MRI exams, 92.4% were performed within 2 weeks in France versus 45% in Québec. Indeed, 40.3% of respondents were able to perform them within 1 week in France, as opposed to 5% in Québec (*p* = 0.002) and 52.1% within 1 to 2 weeks in France and 40% in Québec. No preoperative breast exams in France had to wait for more than 3 weeks (*p* = 0.001).

## Discussion

The results of this study provide an interesting overview of breast MRI practices of radiologists in Québec and France, highlighting the differences between these two different organizational health care systems [8, 9].

### Response rate demographics (questions 1 and 2)

This survey achieved a response rate of nearly 30% (170/571) which corresponds to a good response rate within the reasonable expectations of an internet survey, usually achieving below 50% [6, 10].

Contrary to Clauser et al.’s study where more than 50% of European breast radiologists were based in academic centers, most of the respondents in our study worked instead in non-academic institutions [6]. A comparison of French versus Quebec respondents showed that the majority of French breast radiologists were based in private centers whereas Quebec breast radiologists were mostly based in community hospitals. This reflects the different structural organizations between both health care systems, since the private medical sector in Quebec is frequently “entirely” private as opposed to the French system which is still affiliated with the public health system (not “stricto sensu” private) [8, 9]. As a result, only a minority of breast radiologists in Quebec are based in private institutions and most are practicing in community hospitals, as was noted in our survey.

### Breast MRI examination numbers, indications, and factors influencing recommendation of preoperative breast MRI (questions 3, 4, and 12)

A significant number of respondents in Québec (38%) were not performing any breast MRI as opposed to 2.3 % of respondents in France. A different organizational pattern might explain these numbers. Whereas breast MRIs can be performed in non-“breast” specialized imaging departments in France without specific prerequisite for radiologists interpreting these examinations, breast MRIs are usually performed by radiologists working in specialized centers named Designated Reference Center for Investigations (“Centre de Référence pour Investigation Désigné” (CRID)) in Québec. This type of practice is in accordance with the concept of “Breast Imaging Centers of Excellence” promoted by the American College of Radiology [11].

The number of breast MRI examinations performed by radiologists per week was found to be grossly similar in both countries. Most of the respondents performed 1–10 cases per week, with a substantial number performing 10–21 cases per week in France. These numbers are in agreement with numbers reported in a survey published in 2007 by Bassett, reporting that a majority of respondents (82%) performed 1–15 cases per week in the USA [12].

Indications for performing preoperative breast MRI are still debatable with a recent tendency to decrease preoperative staging breast MRI exams, particularly in the absence of high-level evidence from randomized controlled trials demonstrating benefits of breast MRI in terms of survival [3, 13]. Despite a lack of consensus as noted by several authors [3, 4], criteria employed to recommend preoperative breast MRI exams were consistent both within and also between both countries. Unsurprisingly, in both countries, the three most frequently cited variables considered for breast MRI in patients with newly diagnosed breast cancer were pathologic features, age, and breast density [3, 5]. Of particular note, the surgeon’s opinion was also an important consideration in Québec, much more so than in France. Although this factor has not been extensively evaluated in recent studies [3, 5], it emphasizes the role of breast surgeons in the era of multidisciplinary approach, which is in agreement with Evidence-Based Medicine (EBM) guidelines [14]. Indeed, EBM integrates the best external evidence with individual clinical expertise and the patients’ choices [14]. The role of the surgeon in patient selection for preoperative breast MRI has been recently evaluated by Lee et al. who noted that the decision to perform a preoperative breast MRI is multifactorial and takes into account many patient and tumor-related variables, all of which are weighed at the surgeons’ “discretion” [15]. This significant difference between France and Québec could be explained by their different approach in breast cancer diagnosis, since French breast radiologists have a more extensive and involved role in providing results to patients and performing a preoperative workup before referring the patient to the surgeon [16, 17]. In Québec, on the other hand, particularly in specialized “breast centers,” patients are usually directly referred to the surgeons during the initial diagnosis of breast cancer [18], potentially explaining why the surgeon’s opinion is weighed more heavily. The latter could also partly be related to Quebec’s longer waiting times regarding breast MRI and therefore need to make a clinical decision before performing this examination.

### MRI-guided biopsies (questions 5 and 6)

For nearly 100% of respondents in Québec and France, the estimated performed number of weekly MRI-guided biopsies was reported as 5 or less, which may at first glance appear quite low. However, these numbers are in agreement with a recent multi-centric retrospective French and Swiss study reviewing 1709 MRI-guided biopsies collected over a 7-year period in nine institutions, representing an average number of 27 biopsies per year, per institution [19].

Although essential because of the high sensitivity and limited specificity of breast MRI, MRI-guided biopsies are not widely available in both countries [20, 21]. While some authors consider the limited availability of breast MRI-guided biopsies “a serious weakness” limiting a more generalized usage of this modality [6], the low number of MRI-guided biopsies performed and the extra costs and requirements associated with this procedure (equipment, medical training, time-consuming procedure) could also contribute to explain the situation. In addition, as recommended by the American College of Radiology, an arrangement with affiliated facilities is an acceptable alternative (“In addition, facilities performing breast MRI *must* have the equipment to perform mammographic correlation, directed breast ultrasound, and MRI-guided intervention, or create a referral arrangement with a cooperating facility that could provide these services. The ACR strongly recommends that the cooperating facility be accredited by the ACR in breast MRI.”) [22].

### Waiting time for breast MRI (questions 7, 8, 9, 10, and 11)

Extended wait times endured by patients are a rising and justified concern in our health care systems. This subject has historically been a more sensitive issue in Québec and Canada than in France [23, 24], likely due to the fact that although waiting times are audited in most institutions in both countries, this is more frequently done in Québec (80% versus 68%). Specifically, waiting times for breast MRI exams have been a particular source of concern [3]. Currently, Quebec governmental guidelines recommend a maximal delay of 90 days after reception of the first request whereas French recommendations based on “Plan Cancer 2014–2019” encourage a maximal average delay of 21 days [3, 25]. An audit from 2013 performed at the Centre Hospitalier de l’Université de Montréal showed that among 687 pending breast MRI requests identified at the end of September, 67% exceeded recommended wait times, with 10% exceeding them by more than 12 months [3].

The impact of preoperative MRI on surgical waiting time has been explored through several recent publications [5, 24, 26–28], with all of them, except Vreeland et al. (who does not use time of initial surgery but time of margin-negative surgery as end-point), suggesting that the time from diagnosis to operative treatment of breast cancer has increased over the years, particularly with the advent of breast MRI [5, 26–29]. Although we did not evaluate the impact of preoperative breast MRI on surgical waiting time in our study, our results indicate that breast MRI exams are usually performed within a reasonable time frame following their initial request. Although this delay appears shorter in France, most of the preoperative breast MRI exams in both countries were performed within 3 weeks of their initial request (75% in Québec, 99% in France), which represents an acceptable delay according to the majority of French and Quebec respondents.

Limitations concerning our results that are inherent to survey-based methods must also be taken into account. First, there is always the possibility of selection bias with voluntary respondents who would be more likely to answer if they were particularly interested in the topic at hand. And given that our data collection was performed through an anonymous survey, we have no way of independently confirming or corroborating these self-reported answers. Second, to achieve an acceptable response rate, our questionnaire was also somewhat limited in its ability to precisely ascertain certain variables that may require in depth data collection, such as waiting time for a breast MRI, which was evaluated as an approximate range of weeks, rather than an exact number of days. Third, as our study only addresses pre-MRI waiting time (time from initial request to completion of the preoperative breast MRI examination), we cannot directly comment on the differences regarding preoperative surgical waiting time, which includes both the pre-MRI waiting time and the post-MRI waiting time (time from completion of breast MRI to actual surgery). Given that Zhang et al. have shown that differences in surgical waiting time were most attributable to post-MRI waiting time, due to post-MRI procedures and imaging (second look US and MRI-guided biopsies), this may be an interesting variable to evaluate in future studies given its reported decrease in patient quality of life from additional anxiety [5, 27, 30–32]. Finally, this survey was conducted in 2015–2016, and the responses could have changed over time.

## Conclusion

Overall, our study demonstrates that both Quebec and France make effective use of breast MRI within the confines of their respective health systems. Most radiologists performing breast MRIs work in non-academic institutions and interpret 1–10 breast MRI exams per week. MRI-guided breast biopsies are not widely available in both countries. Quebec demonstrates a higher waiting time than France for breast MRI exams, but most of preoperative breast MRI are performed within 3 weeks in both countries. Of note is the importance of the surgeon’s opinion influencing the recommendation of preoperative MRI in Quebec.

## Data Availability

Data are presented in the “Results” section (tables).

## Abbreviations

EBM: Evidence-Based Medicine
MRI: Magnetic resonance imaging
SCFR: Société Canadienne Française de Radiologie
SIFEM: Société d’Imagerie de la Femme

## References

[1] Gilles R, Guinebretière JM, Lucidarme O (1994). Nonpalpable breast tumors: diagnosis with contrast-enhanced subtraction dynamic MR imaging. Radiology.

[2] Lehman CD, DeMartini W, Anderson BO, Edge SB (2009). Indications for breast MRI in the patient with newly diagnosed breast cancer. J Natl Compr Cancer Netw.

[3] Tan S, David J, Lalonde L (2017). Breast magnetic resonance imaging: are those who need it getting it?. Curr Oncol..

[4] Parsyan A, Moldoveanu D, Balram B (2016). Influence of preoperative magnetic resonance imaging on the surgical management of breast cancer patients. Am J Surg..

[5] Zhang M, Sun S, Mesurolle B (2017). The impact of pre-operative breast MRI on surgical waiting time. PLoS One.

[6] Clauser P, Mann R, Athanasiou A (2018). A survey by the European Society of Breast Imaging on the utilisation of breast MRI in clinical practice. Eur Radiol.

[7] Adkisson CD, Vallow LA, Kowalchik K (2011). Patient age and preoperative breast MRI in women with breast cancer: biopsy and surgical implications. Ann Surg Oncol.

[8] Deber RA (2003). Rekindling reform: lessons from Canada. Am J Public Health.

[9] Rodwin VG (2003). The health care system under French National Health Insurance: lessons for health reform. Am J Public Health.

[10] Thomassin-Naggara I, Jalaguier-Coudray A, Chopier J, Tardivon A, Trop I (2013). Current opinion on clip placement after breast biopsy: a survey of practising radiologists in France and Quebec. Clin Radiol.

[11] American College of Radiology (2013) Breast Imaging Center of Excellence Requirements https://www.acraccreditation.org/~/media/ACRAccreditation/Documents/BICOE/BICOErequirements.pdf?la=en Accessed May 27, 2018

[12] Bassett LW, Dhaliwal SG, Eradat J (2008). National trends and practices in breast MRI. AJR Am J Roentgenol.

[13] Houssami N, Turner R, Morrow M (2013). Preoperative magnetic resonance imaging in breast cancer: meta-analysis of surgical outcomes. Ann Surg.

[14] Sackett DL, Rosenberg WM, Gray JA, Haynes RB, Richardson WS (1996). Evidence based medicine: what it is and what it isn’t. BMJ.

[15] Lee J, Tanaka E, Eby PR (2017). Preoperative breast MRI: surgeons’ patient selection patterns and potential bias in outcomes analyses. AJR Am J Roentgenol.

[16] Boisserie-Lacroix M (2006). Disclosing a diagnosis of breast cancer: what is the role of the radiologist?. J Radiol.

[17] Séradour B, Ancelle-Park R (2006). Breast cancer screening: are results of French and international programmes comparable?. J Radiol.

[18] Dehn T (2008). Who should run breast clinics, surgeons or radiologists?. Ann R Coll Surg Engl.

[19] Verheyden C, Pages-Bouic E, Balleyguier C et al (2016) Underestimation Rate at MR Imaging-guided Vacuumassisted Breast Biopsy: A Multi-Institutional Retrospective Study of 1509 Breast Biopsies. Radiology 281:708-719. 10.1148/radiol.2016151947 27355898

[20] Plantade R, Thomassin-Naggara I (2014). MRI vacuum-assisted breast biopsies. Diagn Interv Imaging.

[21] Chopier J, Dratwa C, Antoine M, Gonin J, Thomassin Naggara I (2014). Radiopathological correlations: masses, non-masslike enhancements and MRI-guidedbiopsy. Diagn Interv Imaging.

[22] https://www.acraccreditation.org/~/media/ACRAccreditation/Documents/Breast-MRI/Requirements.pdf Accessed May 27, 2018

[23] Barua B (2017) Waiting Your Turn: Wait Times for Health Care in Canada, 2017 Report. https://www.fraserinstitute.org/studies/waiting-yourturn-wait-times-forhealth-care-in-canada-2017 Accessed July 31, 2018

[24] Molinie F, Leux C, Delafosse P (2013). Waiting time disparities in breast cancer diagnosis and treatment: a population-based study in France. Breast.

[25] Plan Cancer 2014-2019. http://solidarites-sante.gouv.fr/IMG/pdf/2014-02-03_Plan_cancer-2.pdf Accessed July 30, 2018

[26] Hulvat M, Sandalow N, Rademaker A, Helenowski I, Hansen NM (2010). Time from diagnosis to definitive operative treatment of operable breast cancer in the era of multimodal imaging. Surgery.

[27] Kothari A, Fentiman IS (2003) 22. Diagnostic delays in breast cancer and impact on survival. Int J Clin Pract 57:200–203 12723724

[28] Bleicher RJ, Ciocca RM, Egleston BL (2009). Association of routine pretreatment magnetic resonance imaging with time to surgery, mastectomy rate, and margin status. J Am Coll Surg.

[29] Vreeland TJ, Berry Iv JS, Schneble E et al (2017) Routine pre-treatment MRI for breast cancer in a single-payer medical center: effects on surgical choices, timing and outcomes J Cancer 23; 8:2442-2448 10.7150/jca.16738 PMC5595073 28900481

[30] Flory N, Lang EV (2011). Distress in the radiology waiting room. Radiology.

[31] Lang EV, Berbaum KS, Lutgendorf SK (2009). Large-core breast biopsy: abnormal salivary cortisol profiles associated with uncertainty of diagnosis. Radiology.

[32] Brocken P, Prins JB, Dekhuijzen PN, van der Heijden HF (2012). The faster the better?-A systematic review on distress in the diagnostic phase of suspected cancer, and the influence of rapid diagnostic pathways. Psychooncology.

